# Supplementary material for: An anisotropic strategy for developing polymer electrolytes endowing lithium metal batteries with electrochemo-mechanically stable interface
Source: Nat Commun. 2025 Apr 16;16:3626. doi: 10.1038/s41467-025-58916-x (PMC12003723; doi:10.1038/s41467-025-58916-x)
Supplement: Supplementary file 1 — Supporting Information [file 41467_2025_58916_MOESM1_ESM.pdf]

An Anisotropic Strategy for Developing Composite Polymer Electrolytes Endowing Lithium Metal Batteries with Electrochemo-mechanically Stable Interface

Jingren Gou[1,5], Kaixuan Cui[1,5], Suqing Wang[2✉], Zheng Zhang[1,3✉], Jiale Huang[4], Haihui Wang[1✉]

[1]Dr. J. Gou, Dr. K. Cui, Dr. Z. Zhang, Prof. H. Wang,

State Key Laboratory of Chemical Engineering, Department of Chemical Engineering, Tsinghua University, Beijing 100084, China.

[2]Prof. S. Wang

School of Chemistry and Chemical Engineering, South China University of Technology, Guangzhou 510640, China

[3]Dr. Z. Zhang

State Key Laboratory of Efficient Production of Forest Resources, Beijing Key Laboratory of Lignocellulosic Chemistry, Beijing Forestry University, Beijing 100083, China

[4]Dr. J. Huang

School of Mechanical and Electrical Engineering, Guangzhou University, Guangzhou 510000, China

[5]These authors contributed equally: Jingren Gou, Kaixuan Cui

✉**e-mail:**cesqwang@scut.edu.cn; zhengzhang0527@163.com**;** cehhwang@tsinghua.edu.cn**;**

Table of Contents

1. Supplementary Figures 1-32
2. Supplementary Tables 1-8
3. Supplementary References 1-14

1. Supplementary Figures 1-32

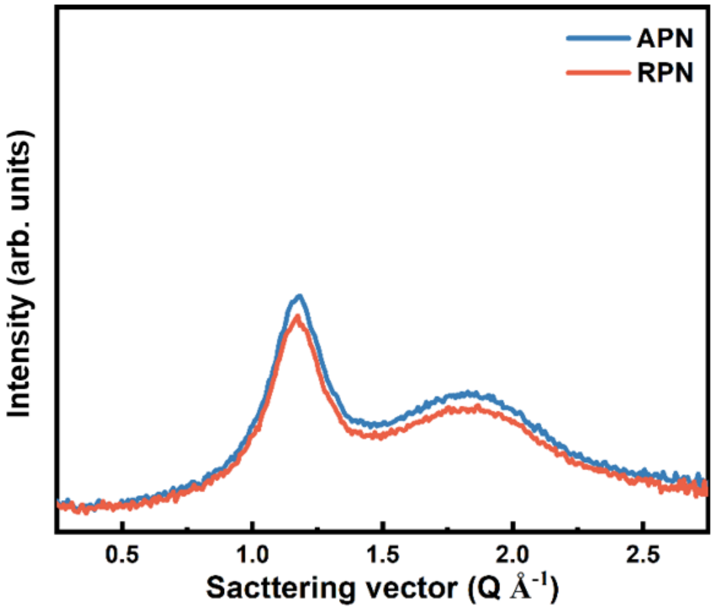

Supplementary Figure 1. 1D WAXS plots of APN and RPN.

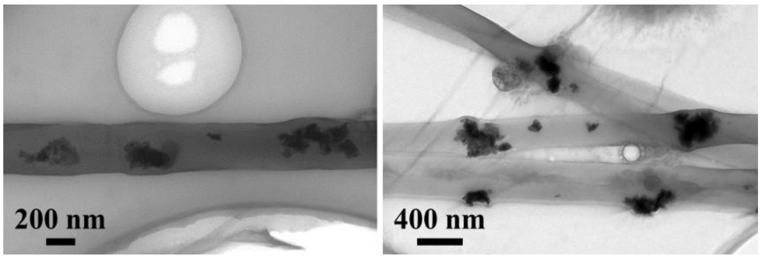

Supplementary Figure 2. The TEM images of the nanofiber in LAPN.

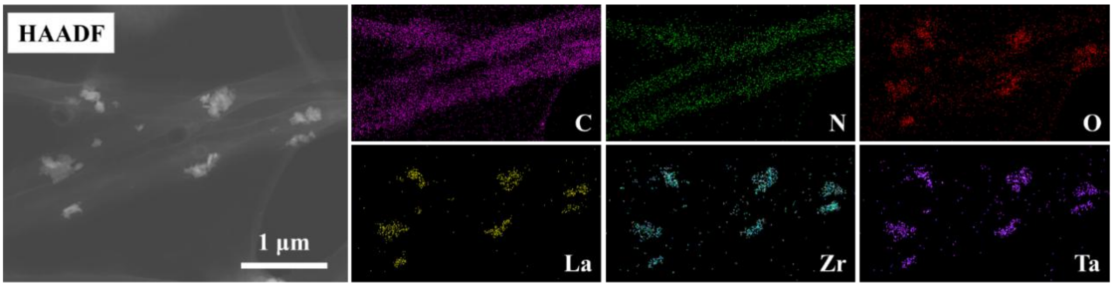

Supplementary Figure 3. The EDS mapping of the nanofibers in LAPN.

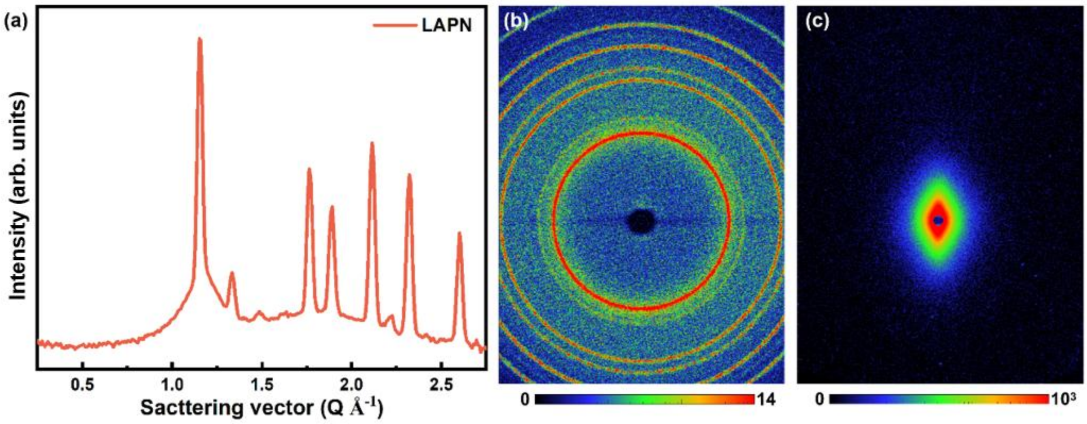

Supplementary Figure 4. (**a**) 1D WAXS plot of LAPN. (**b**) 2D WAXS and (**c**) 2D SAXS plots of LAPN.

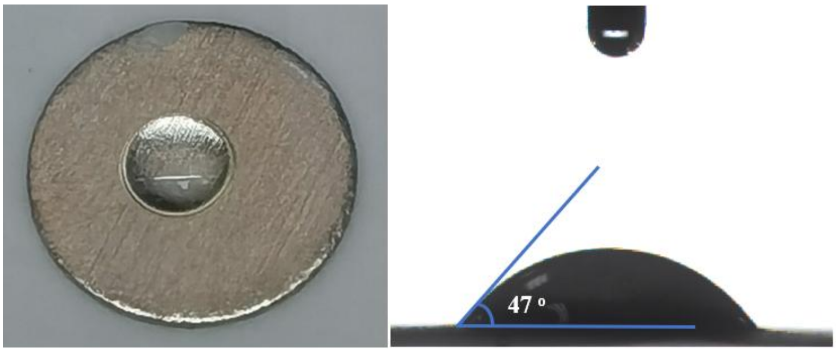

Supplementary Figure 5. The contact angle measurement of the polymer precusor on the lithium metal.

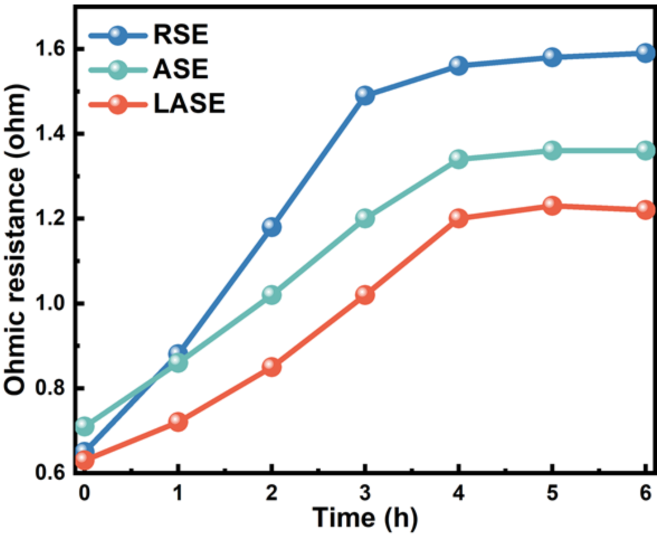

Supplementary Figure 6. Ohmic resistance of SS||SS cells as a function of time when heated at 70 °C.

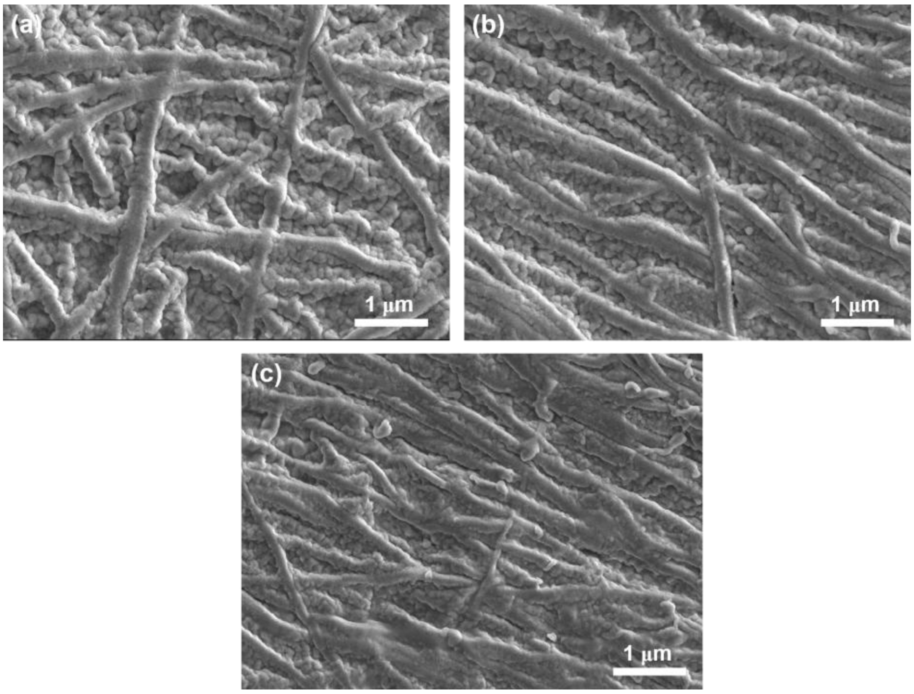

Supplementary Figure 7. SEM image of (**a**) RSE, (**b**) ASE and (**c**) LASE from the top view.

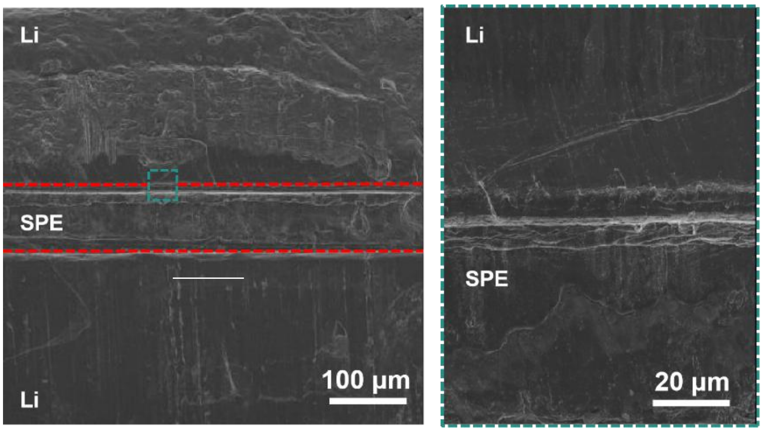

Supplementary Figure 8. The SEM image of the Li/Li symmetrical cell.

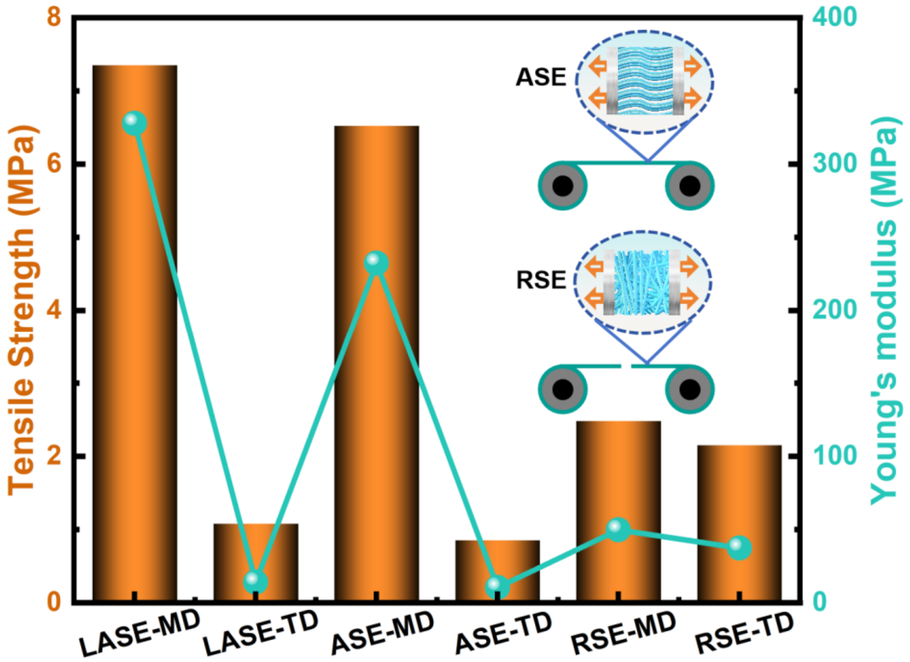

Supplementary Figure 9. Tensile strength and Young's modulus of RSE, ASE and LASE at MD and TD.

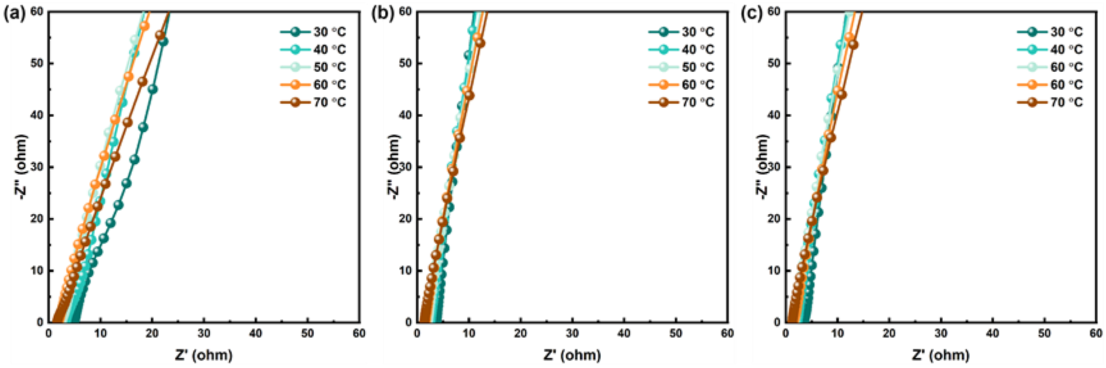

Supplementary Figure 10. Temperature-dependent EIS plots of (**a**) RSE, (**b**) ASE and (**c**) LASE.

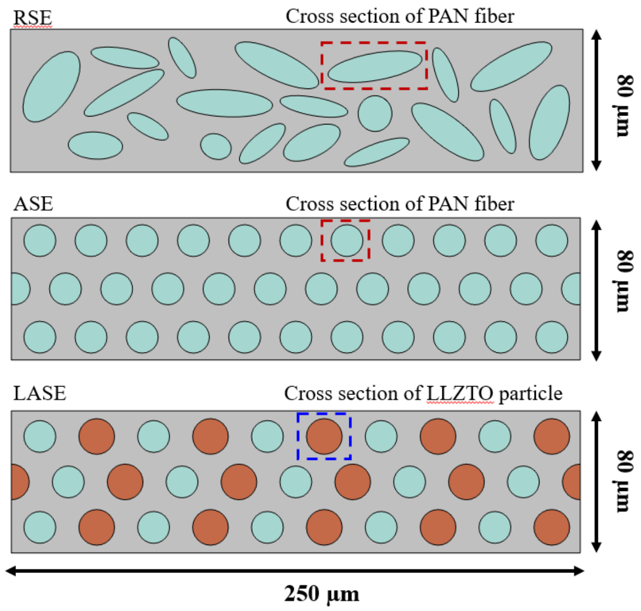

Supplementary Figure 11. Schematic demonstration of the 2D diffusion model of RSE, ASE, and LASE.

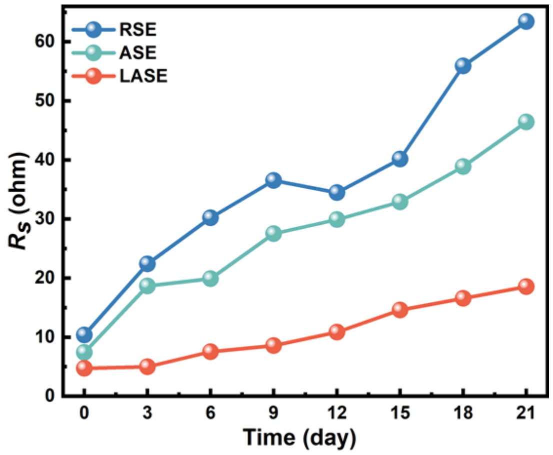

Supplementary Figure 12. Temporal variation of R_s of Li||Li cells.

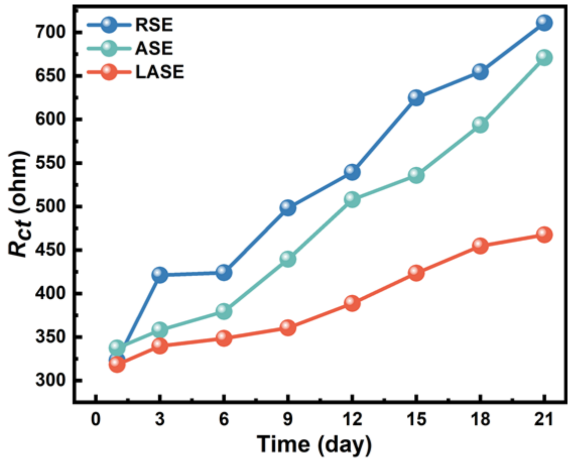

Supplementary Figure 13. Temporal variation of R_{ct} of Li||Li cells.

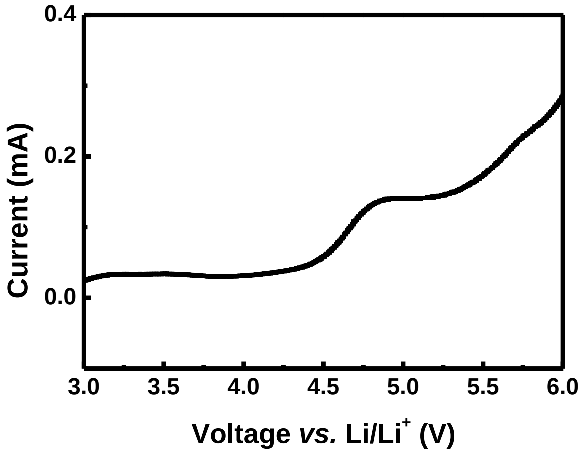

Supplementary Figure 14. LSV curve to characterize the electrochemical window of PEGDA.

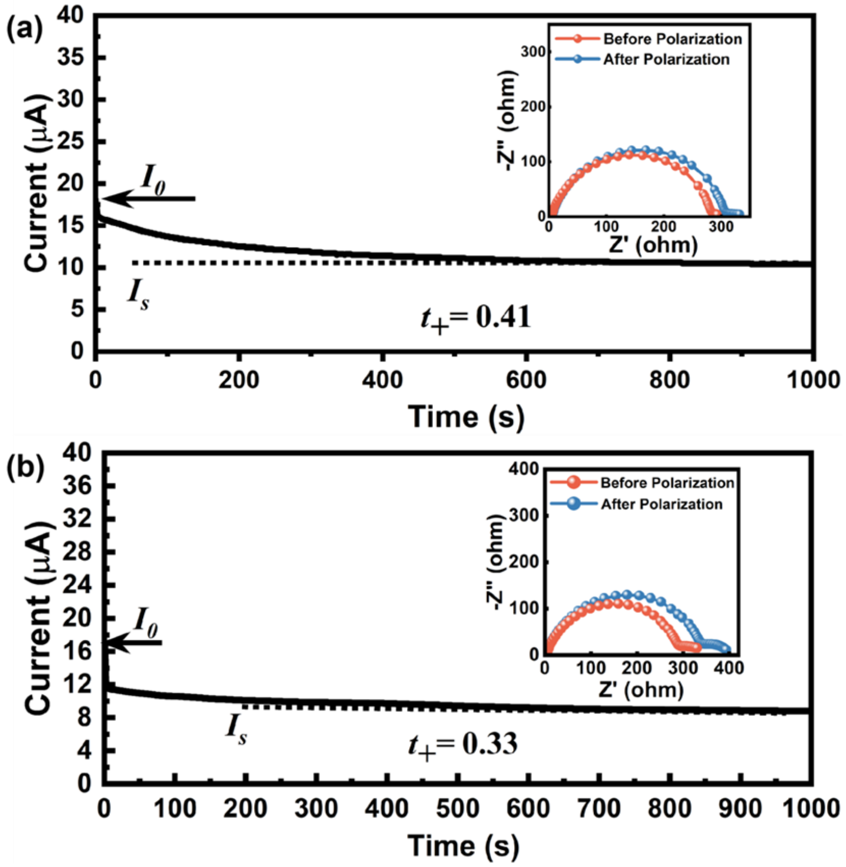

Supplementary Figure 15. Chronoamperometry and EIS results to calculate t_+ of (**a**) ASE and (**b**) RSE.

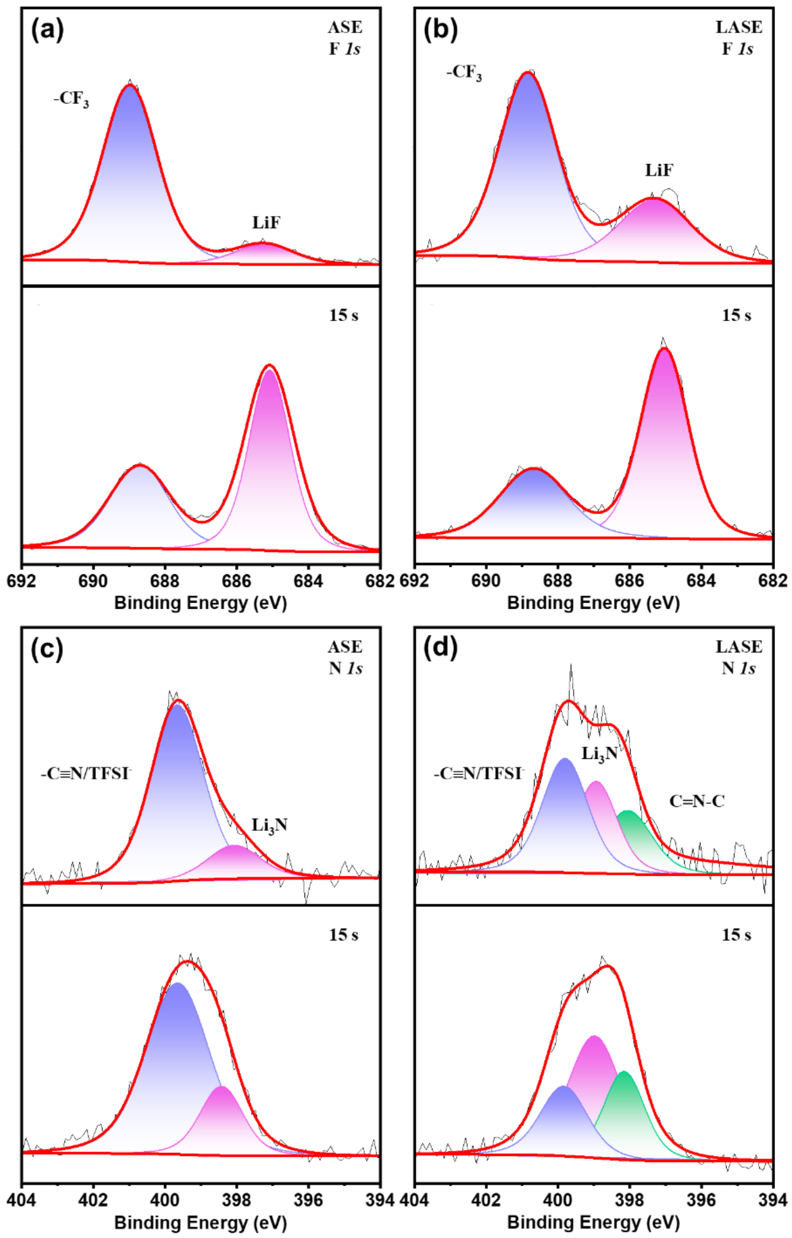

Supplementary Figure 16. XPS spectra to analyze the chemical composition of the Li anode cycled with ASE and LASE: (a,b) F *1s* and (c,d) N *1s*.

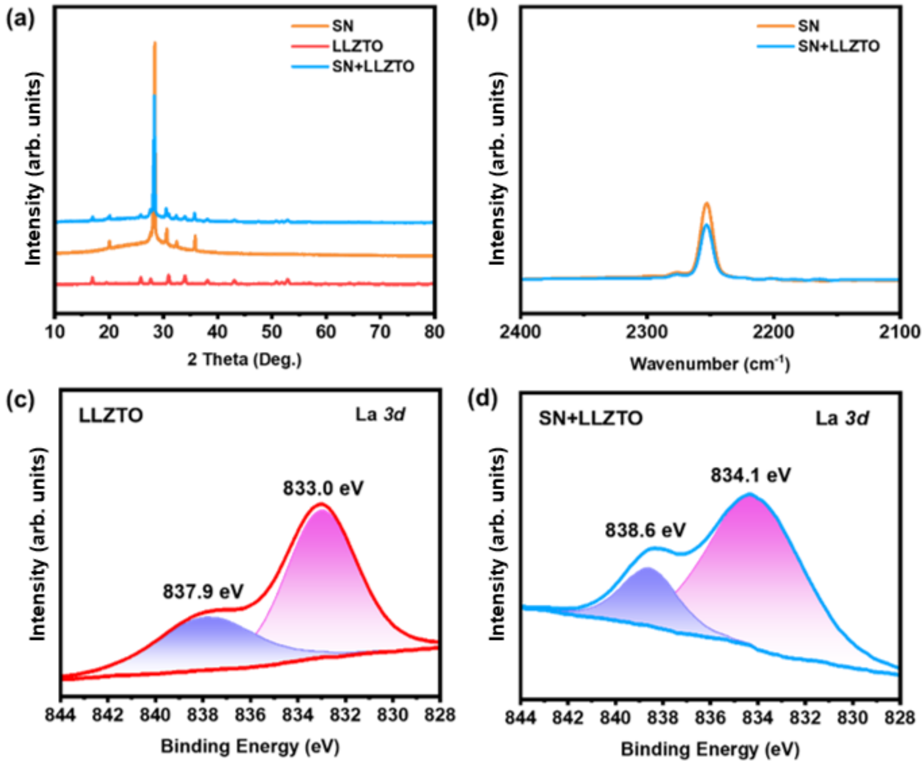

Supplementary Figure 17. (a) XRD patterns of SN, LLZTO and SN+LLZTO. (b) FTIR spectra of SN and SN +LLZTO. XPS spectra of (c) LLZTO and (d) SN +LLZTO.

Initially, the chemical compatibility between LLZTO and SN was examined using XRD. As depicted in Supplementary Figure 17a, the characteristic peaks remained unchanged after mixing LLZTO with SN, indicating a good chemical compatibility. Subsequently, FTIR was conducted on SN and SN+LLZTO to explore the interaction between LLZTO and SN (Supplementary Figure 17b). The vibrational peak observed at 2252.9 cm^{-1} corresponds to the C≡N stretching mode of SN, and the decrease in peak intensity upon the addition of LLZTO may be due to the interaction between the C≡N groups of SN and LLZTO. Further analysis of the electronic aggregation state of LLZTO in the SN+LLZTO mixture was performed using XPS, and the results are shown in Supplementary Figure 17c-d. The positive shift observed in the La *3d* spectra after the addition of SN indicates an increase in the binding energy of La 3*d*. This may be attributed to the interaction between La atoms (in LLZTO) and N atoms (in SN), leading to a reduction in the electronic density around the La atoms.

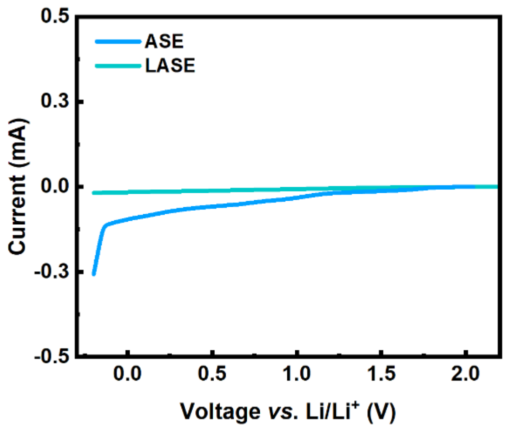

Supplementary Figure 18. LSV curves of the cells using ASE and LASE at a scan rate of 1 mV·s^{-1} at the potential range from open circuit voltage to -0.2 V.

As shown in **Supplementary Figure 18**, the LASE electrolyte demonstrates superior resistance to reduction compared with ASE, indicating that the addtion of LLZTO in LASE can contribute to enhancing the interfacial stability with the Li anode.

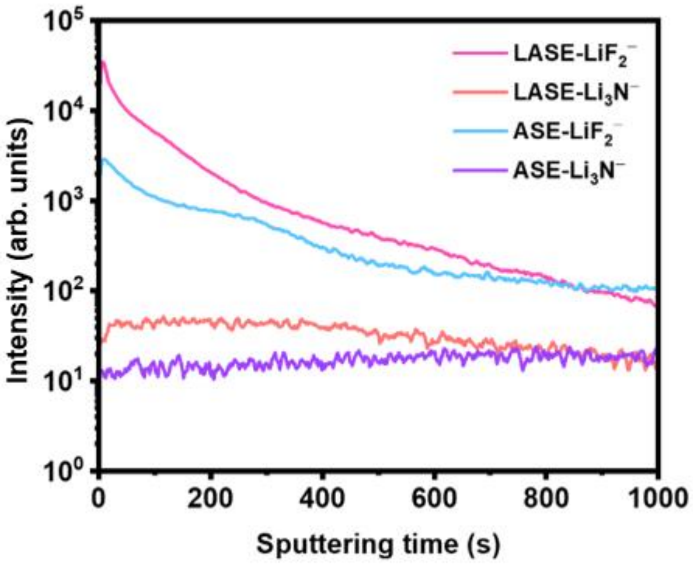

Supplementary Figure 19. ToF-SIMS depth profiles of various atom group fragments formed on the Li anode after cycling.

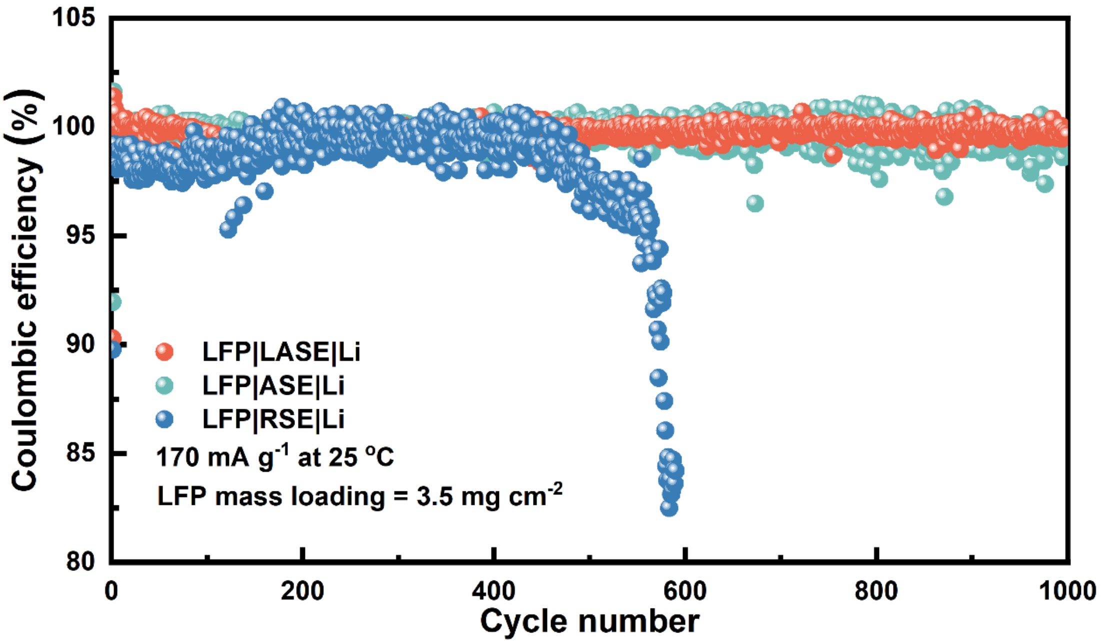

Supplementary Figure 20. The coulombic efficiency of Li||LFP cells with the LFP mass loading of 3.5 mg cm^{-2}.

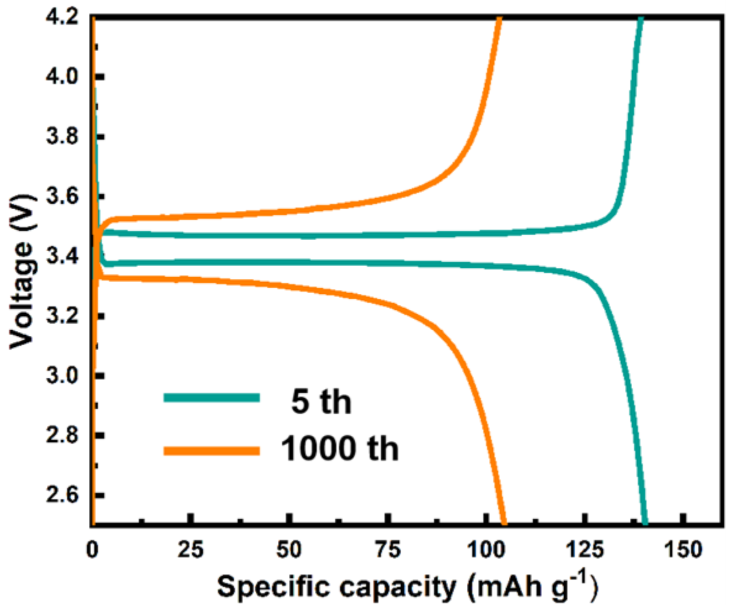

Supplementary Figure 21. Voltage profiles of Li|ASE|LFP with the low mass loading cathode at the initial and final stage of the cycling test.

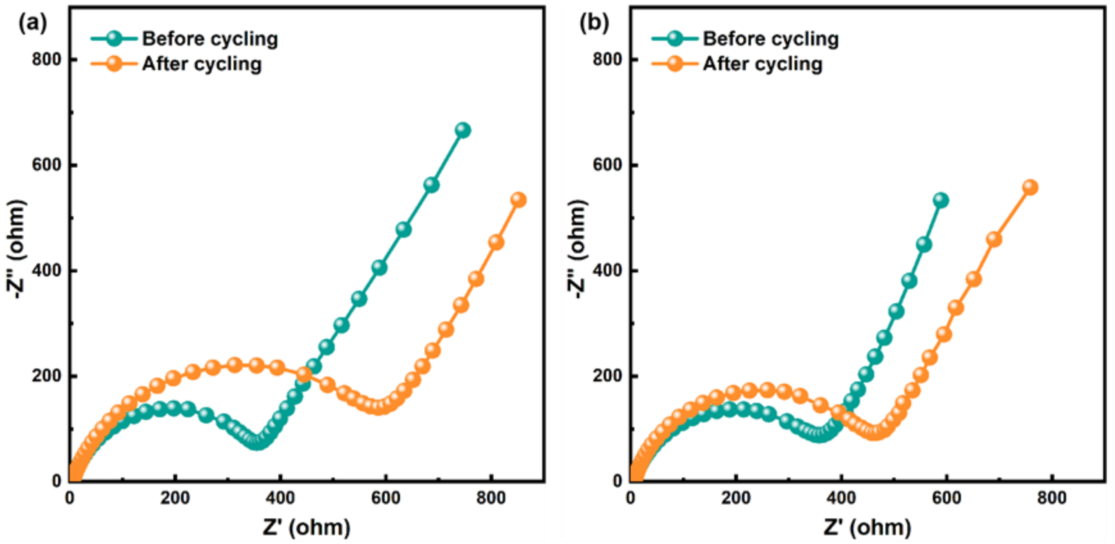

Supplementary Figure 22. EIS plots of Li||LFP assembled with (**a**) RSE and (**b**) ASE before and after the cycling test.

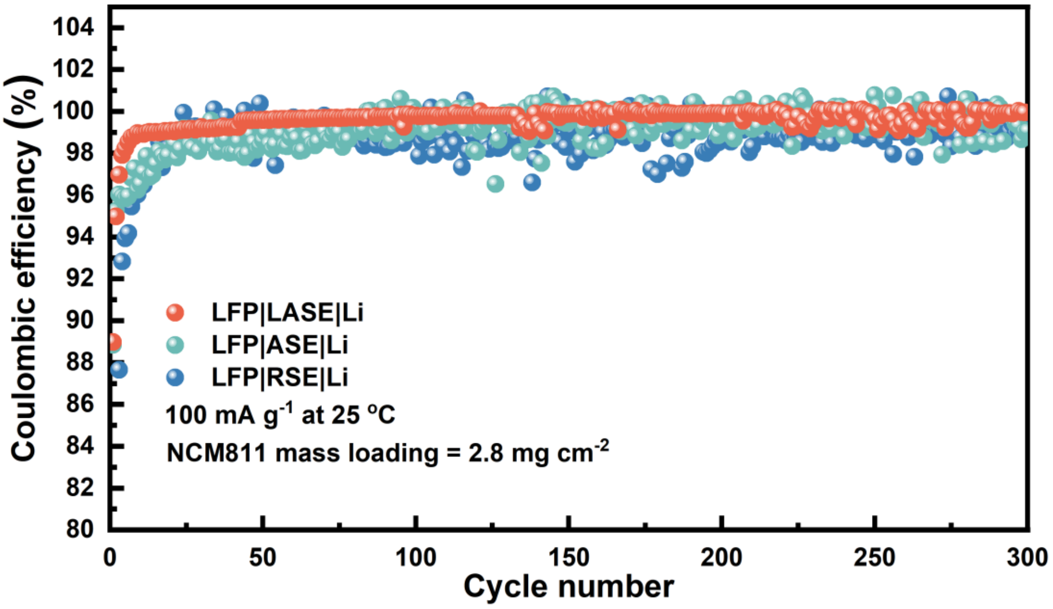

Supplementary Figure 23. The coulombic efficiency of Li||NCM811 cells with the NCM811 mass loading of 2.5 mg cm^{-2}.

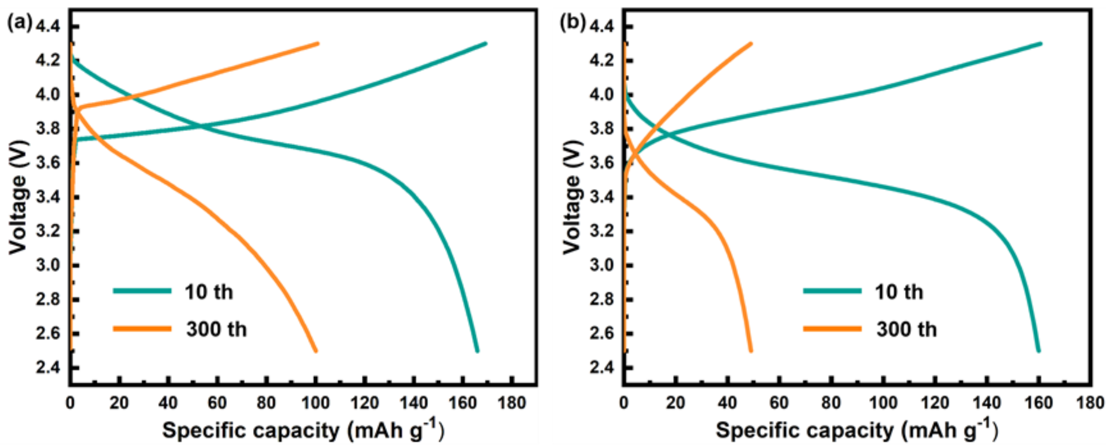

Supplementary Figure 24. Voltage profiles of Li||NCM811 assembled with (**a**) ASE and (**b**) RSE at the initial and final stages of the cycling test.

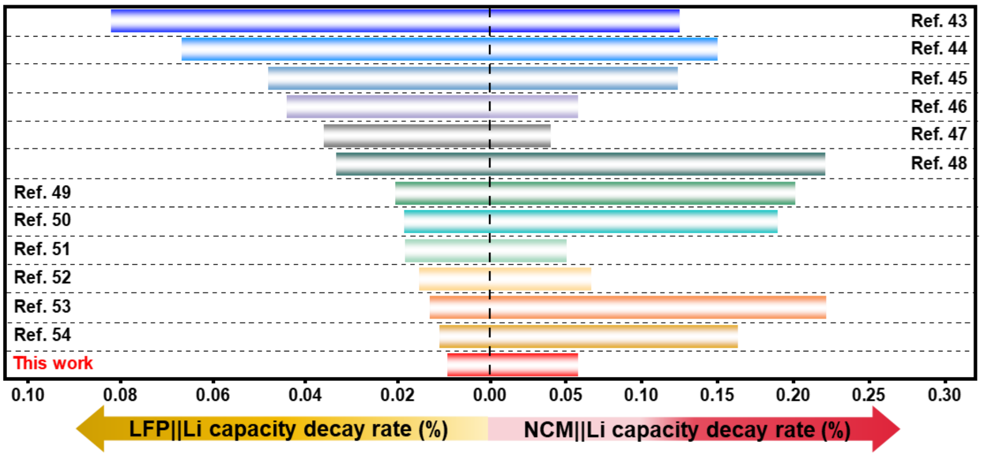

Supplementary Figure 25. Comparison of cycling performances between LASE and other SPEs reported in recent works

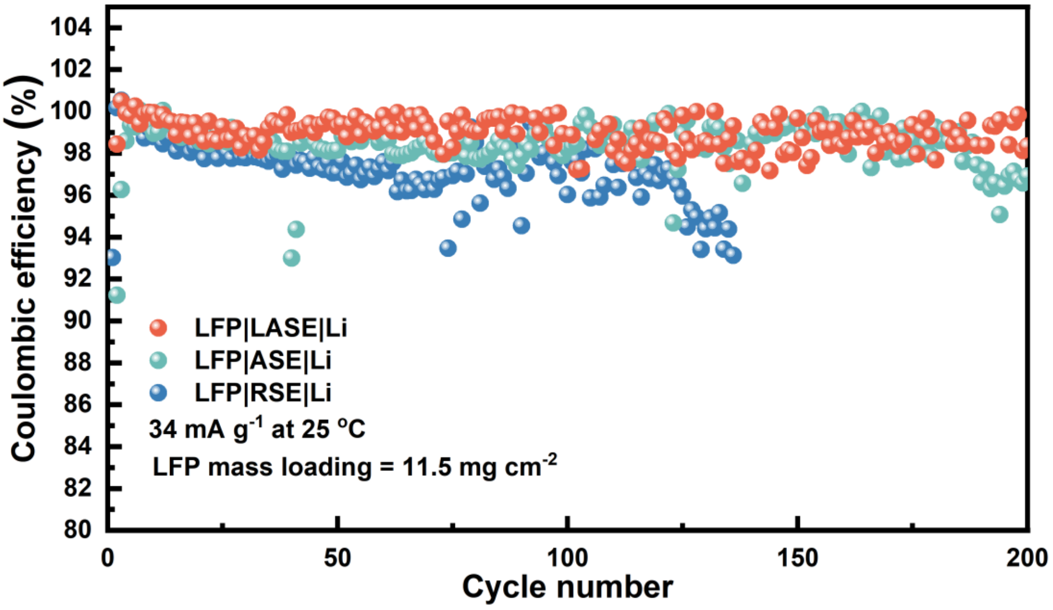

Supplementary Figure 26. The coulombic efficiency of Li||LFP cells with the LFP mass loading of 11.5 mg cm^{-2}.

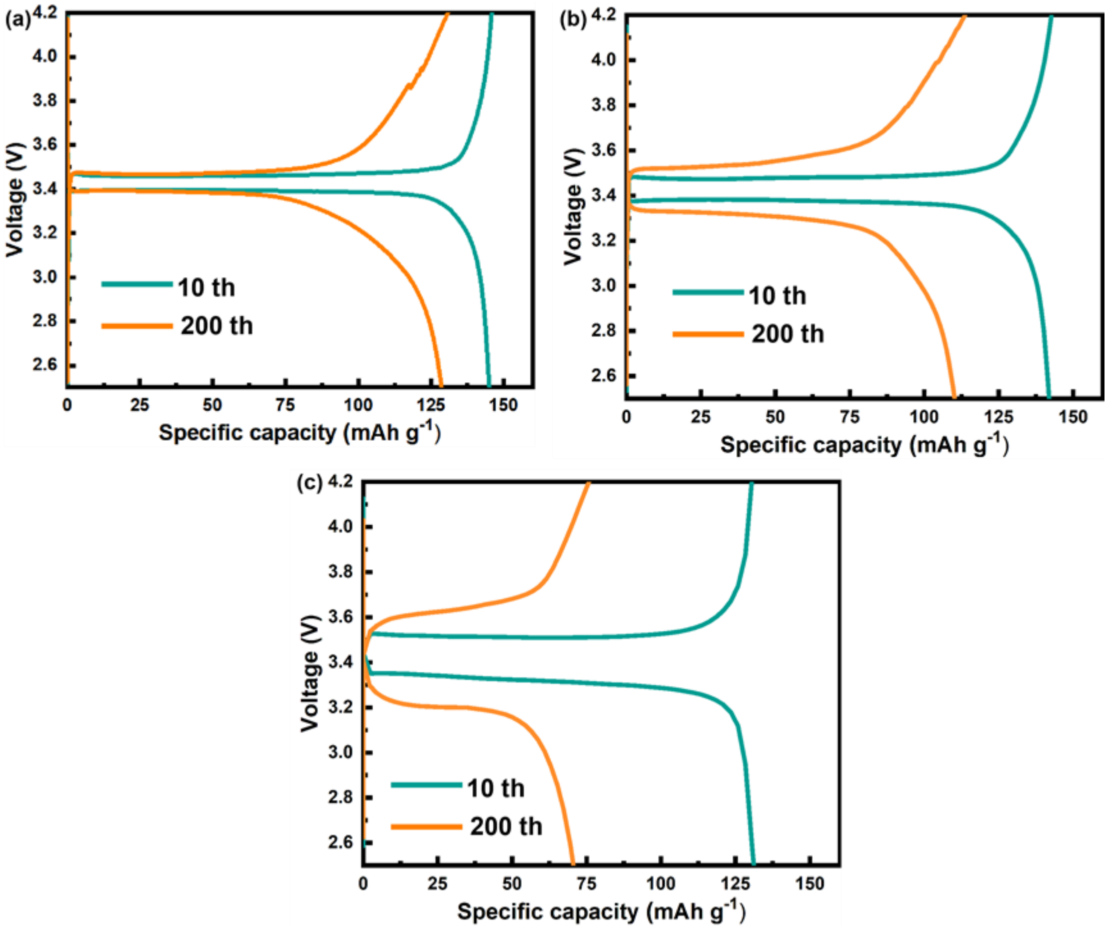

Supplementary Figure 27. Voltage profiles of (**a**) Li|LASE|LFP, (**b**) Li|ASE|LFP , and (**c**) Li|RSE|LFP with the high mass loading cathode at the initial and final stage of the cycling test.

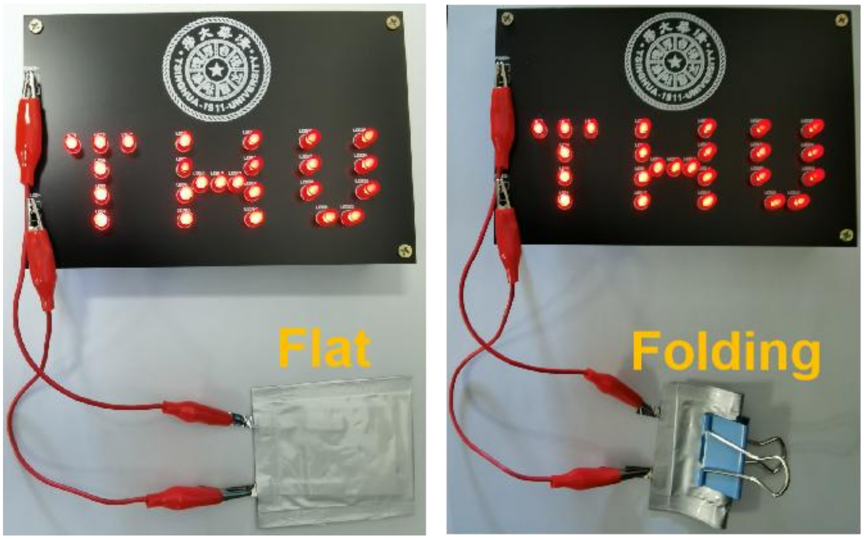

Supplementary Figure 28. The digital photos of NCM811|LASE|Li pouch cell lighting up LED bubbles under flat and folding states.

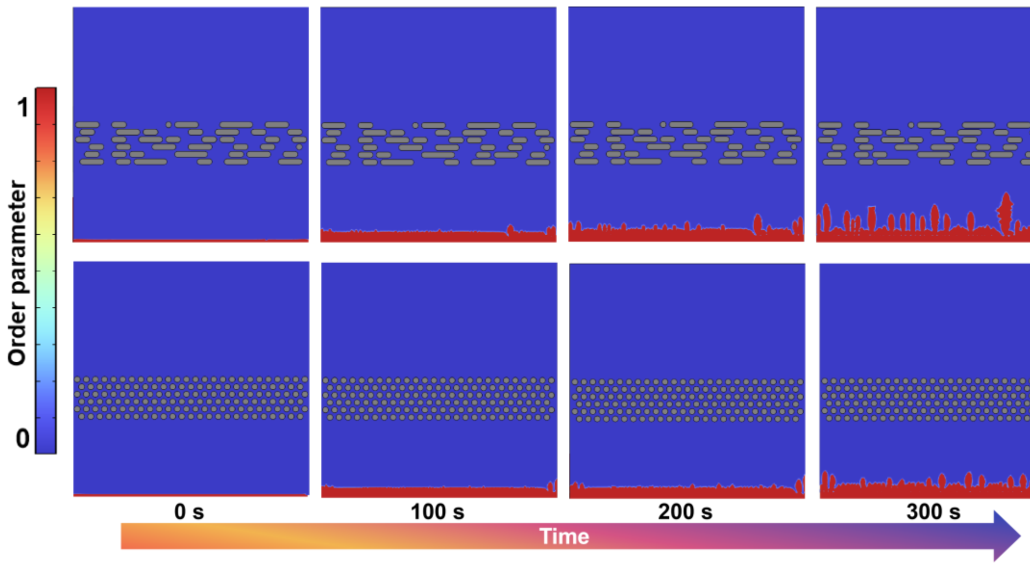

Supplementary Figure 29. Spatial-temporal evolution of Li dendrites modulated by RSE (up) and ASE (down).

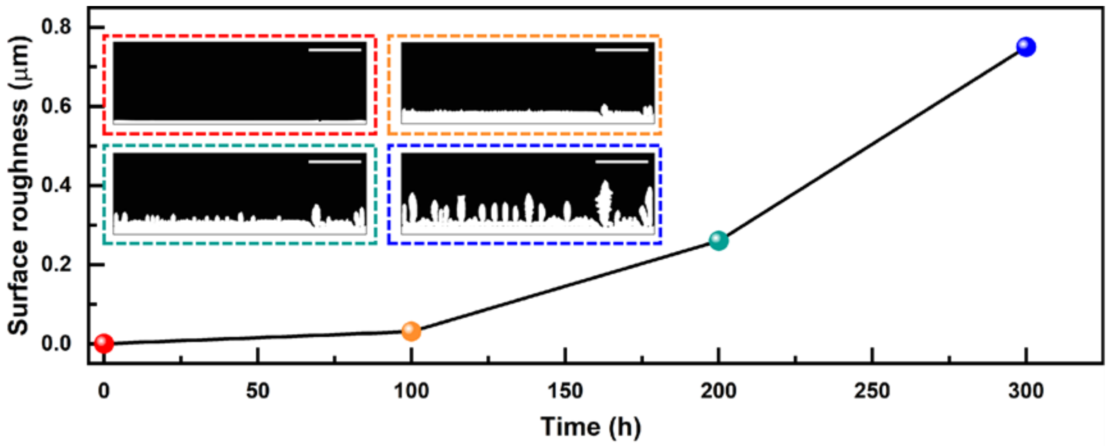

Supplementary Figure 30. Evolution of surface roughness of Li anode surface paired with RSE. Scale bars, 1μm.

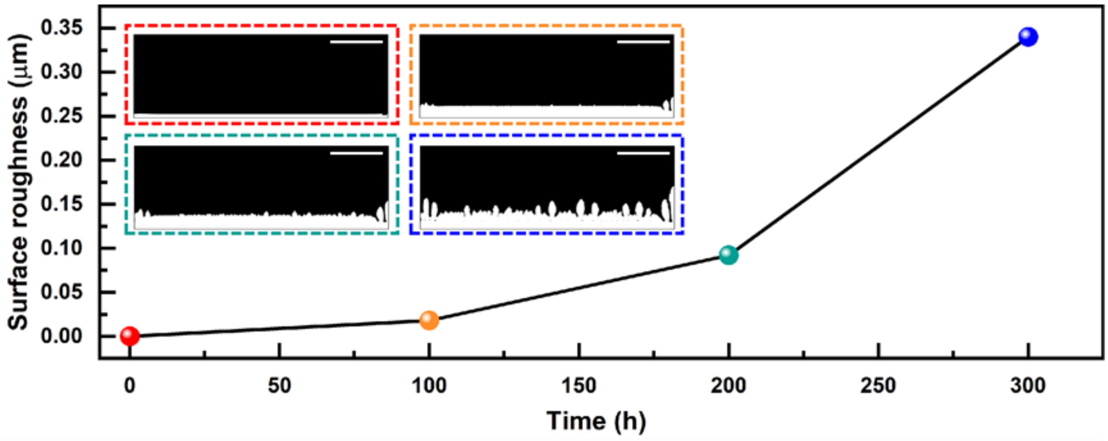

Supplementary Figure 31. Evolution of the surface roughness of the Li anode surface paired with ASE. Scale bars, 1μm.

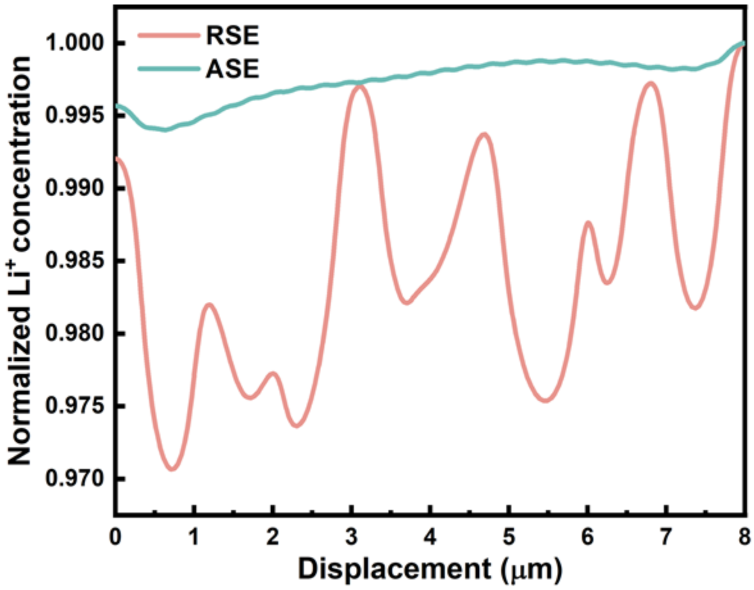

Supplementary Figure 32. Distribution of Li^+ along the vertical cross-section (white dash lines in Figure 5d) adjacent to the supporting network.

2. Supplementary Table 1-8

Supplementary Table 1. Detail parameters to calculate ionic conductivity σ.

	Thickness (μm)	Contact area (cm^{-2})	R_b at 20 °C (Ω)	σ (mS cm^{-1})
RSE	80.05	1.61	4.70	1.06
ASE	82.55	1.61	3.84	1.33
LASE	86.10	1.61	3.66	1.46

Supplementary Table 2. The values of equivalent circuit elements and fitting errors of EIS plots at different days

Day	Sample	R_{int} (Ω)	R_s (Ω)	R_{ct} (Ω)	Fitting error (%)
1st day	RSE	333.7	10.2	323.5	0.118
	ASE	344.9	7.4	337.5	0.270
	LASE	323.0	4.7	318.3	0.139
3rd day	RSE	443.6	22.4	421.2	0.366
	ASE	376.7	18.7	358.0	0.155
	LASE	344.7	5.0	339.7	0.141
6th day	RSE	454.2	30.2	424.0	0.210
	ASE	399.3	19.9	379.4	0.308
	LASE	356.1	7.5	348.6	0.185
9th day	RSE	535.0	36.5	498.5	0.473
	ASE	466.9	27.5	439.2	0.175
	LASE	369.2	8.6	360.6	0.212
12th day	RSE	573.8	34.4	539.8	0.301
	ASE	537.8	29.9	507.9	0.468
	LASE	399.5	10.8	388.7	0.132
15th day	RSE	665.0	40.1	624.8	0.225
	ASE	568.5	32.9	535.2	0.398
	LASE	438.0	14.6	423.5	0.265
18th day	RSE	710.7	55.9	654.8	0.553
	ASE	632.5	38.8	593.6	0.670
	LASE	471.1	16.6	454.5	0.382
21th day	RSE	774.3	63.4	710.9	0.485
	ASE	717.4	46.4	671.0	0.510
	LASE	485.9	18.5	467.4	0.244

Supplementary Table 3. Detail parameters to calculate Li^+ transference number

	I_0 (μA)	I_s (μA)	R_0 (Ω)	R_s (Ω)	ΔV (V)
RSE	17.04	8.21	286.37	293.12	0.01
ASE	18.08	10.05	279.66	316.44	0.01
LASE	21.31	16.01	293.65	341.20	0.01

Supplementary Table 4. The comparison of RSE, ASE and LASE.

	Cycling lifespan of Li|Li cells (h)	Electrochemical window (V)	Ionic conductivity (mS cm^{-1})	Li^+ transference number	Tensile Strength (MPa)
RSE	240	4.7	1.06	293.12	2.48
ASE	750	4.7	1.33	316.44	6.52
LASE	1500	4.8	1.46	341.20	7.35

Supplementary Table 5. Average capacity decay comparison of LASE with recent publications.

Electrolyte	Li||LFP decay rate (%)/Cycle number	Li||NCM decay rate (%)/Cycle number	Temperature
PEO-5LiMPS[1]	0.082/200	0.125/200	30 °C
NDCPE-5%[2]	0.0666667/300	0.15/200	25 °C
PTADOL[3]	0.048/300	0.124/150	25 °C
PEO/CuF_2[4]	0.044/500	0.058/120	30 °C
Cellulose/B_2O_3[5]	0.036/500	0.04/200	25 °C
PNPU-PVDF-HFP[6]	0.0333333/300	0.221/100	25 °C
PPL-PVCL[7]	0.0204333/300	0.201/100	25 °C
PEO-01PS[8]	0.0186923/1300	0.1895/200	-
p-3DSE[9]	0.0183333/600	0.0506667/300	25 °C
SE[10]	0.0153846/1300	0.0666667/300	25 °C
$PAN_{1.2}$-SPE[11]	0.0131333/1500	0.2216667/100	25 °C
SH-SPE[12]	0.0108696/230	0.1633333/120	25 °C
This work (LASE)	0.0092/1000	0.058/300	25 °C

Supplementary Table 6. Calculation of the gravimetric energy density of Li||NCM811 pouch cell.

Component of pouch cell	Parameter	Value
Cathode (NCM811)	Active material loading (mg cm^{-2})	9.80
	Cathode loading (mg cm^{-2})	10.32
	Thickness (μm)	40
ASE	Thickness (μm)	80
	Areal density (mg cm^{-2})	5.78
LASE	Thickness (μm)	80
	Areal density (mg cm^{-2})	6.05
Anode (Li)	Thickness (μm)	40
	Areal density (mg cm^{-2})	2.14
Current collector (Al)	Thickness (μm)	14
	Areal density (mg cm^{-2})	3.78
Pouch cell Li|ASE|NCM811	Total mass (mg cm^{-2})	22.02
	Total thickness (μm)	174
	Areal capacity (mAh cm^{-2})	1.86
	Average discharge voltage (V)	3.744
	Gravimetric energy density (Wh kg^{-1})	316.3
	Volume energy density (Wh L^{-1})	400.2
Pouch cell Li|LASE|NCM811	Total mass (mg cm^{-2})	22.29
	Total thickness (μm)	174
	Areal capacity (mAh cm^{-2})	1.94
	Average discharge voltage (V)	3.751
	Gravimetric energy density (Wh kg^{-1})	326.4
	Volume energy density (Wh L^{-1})	418.2

Supplementary Table 7. Parameters calculated by DFT theory to determine the adsorption energy.

	$E_{ads,SN}$ (eV)	$E_{ads,Li}$ (eV)	$E_{ads,LLZTO}$ (eV)	$E_{ads,SN-Li}$ (eV)	$E_{ads,SN-LLZTO}$ (eV)
SN-Li	-1251.37	-25340.62	-	-26595.54	-
SN-LLZTO	-1251.37	-	-23832.95	-	-25087.12

Supplementary Table 8. Basic parameters related to governing equations.

Parameter	Symbol	Real value
Interfacial mobility	L_i	2.5×10^{-6} m^3 J^{-1} s^{-1} [13, 14]
Barrier height	W	3.75×10^5 J m^{-3} [13, 14]
Reaction coefficient	L_m	1 s^{-1} [13, 14]
Gradient energy coefficient	κ_0	2.5×10^{-5} J m^{-1} [13, 14]
Diffusivity in electrode	De	7.5×10^{-13} m^2 s^{-1} [13, 14]
Diffusivity in electrolyte	D_l	2.5×10^{-10} m^2 s^{-1} [13, 14]
Conductivity in electrode	S_e	10^7 S m^{-1} [13, 14]
Conductivity in electrolyte	S_l	1 S m^{-1} [13, 14]
Symmetrical factors	α, β	0.5[13, 14]
Strength of the anisotropy	ω	4[13, 14]
Mode of anisotropy	δ	0.05[13, 14]
Temperature	T	298.15 K

3.Supplementary References 1-14

1. Jiang B*, et al.* Polymer electrolytes shielded by 2D $Li_{0.46}Mn_{0.77}PS_3$ Li^+-conductors for all-solid-state lithium-metal batteries. *Energy Storage Mater.* **56**, 183-191 (2023).

2. Lv S*, et al.* A Supertough and Highly-Conductive Nano-Dipole Doped Composite Polymer Electrolyte with Hybrid Li^+-Solvation Microenvironment for Lithium Metal Batteries. *Adv. Energy Mater.* **13**, 2302711 (2023).

3. Du Y*, et al.* Ameliorating structural and electrochemical properties of traditional poly-dioxolane electrolytes via integrated design of ultra-stable network for solid-state batteries. *Energy Storage Mater.* **56**, 310-318 (2023).

4. Wei Y*, et al.* Enabling All-Solid-State Li Metal Batteries Operated at 30 °C by Molecular Regulation of Polymer Electrolyte. *Adv. Energy Mater.* **13**, 2203547 (2023).

5. Cheng H*, et al.* Inorganic-Rich Interphase Induced by Boric Oxide Solid Acid toward Long Cyclic Solid-State Lithium-Metal Batteries. *Adv. Funct. Mater.* **34**, 2307677 (2023).

6. Ye Y, Zhu X, Meng N, Lian F. Largely Promoted Mechano-Electrochemical Coupling Properties of Solid Polymer Electrolytes by Introducing Hydrogen Bonds-Rich Network. *Adv Funct. Mater.* **33**, 2307045 (2023).

7. Zheng J*, et al.* Heterocyclic polymer supported cathode/Li interface layers to lower the operational temperature of PEO-based Li-batteries. *Nano Energy* **118**, 108975 (2023).

8. Zhang J*, et al.* Construction of Stable Li_2O-Rich Solid Electrolyte Interphase for Practical PEO-Based Li-Metal Batteries. *Adv. Energy Mater.* **14**, 2302587 (2023).

9. Mu Y*, et al.* Highly Efficient Aligned Ion-Conducting Network and Interface Chemistries for Depolarized All-Solid-State Lithium Metal Batteries. *Nano-micro Lett.* **16**, 86-86 (2024).

10. Lee J, Park H, Hwang J, Noh J, Yu C. Delocalized Lithium Ion Flux by Solid-State Electrolyte Composites Coupled with 3D Porous Nanostructures for Highly Stable Lithium Metal Batteries. *ACS Nano* **17**, 16020-16035 (2023).

11. Zhang D*, et al.* Eutectic-Based Polymer Electrolyte with the Enhanced Lithium Salt Dissociation for High-Performance Lithium Metal Batteries. *Angew. Chem. Int. Edit.* **62**, e202310006 (2023).

12. Zhao L*, et al.* Dynamic Supramolecular Polymer Electrolyte to Boost Ion Transport Kinetics and Interfacial Stability for Solid-State Batteries. *Adv. Funct. Mater.* **33**, 2214881 (2023).

13. Chen L*, et al.* Modulation of dendritic patterns during electrodeposition: A nonlinear phase-field model. *J. Power Sources* **300**, 376-385 (2015).

14. Shen X, Zhang R, Shi P, Chen X, Zhang Q. How Does External Pressure Shape Li Dendrites in Li Metal Batteries? *Adv. Funct. Mater.* **11**, 2003416 (2021)
