# Supplementary material for: An anisotropic strategy for developing polymer electrolytes endowing lithium metal batteries with electrochemo-mechanically stable interface
Source: Nat Commun. 2025 Apr 16;16:3626. doi: 10.1038/s41467-025-58916-x (PMC12003723; doi:10.1038/s41467-025-58916-x)
Supplement: Supplementary file 2 — Description of Additional Supplementary Files [file 41467_2025_58916_MOESM2_ESM.pdf]

Description of Additional Supplementary Files

Supplementary Data 1. The atomic coordinates of optimized absorption models of Li-SN and LLZTO-SN reported in the manuscript.
